# Metals Can Change the Colors of Eggshells but How Is This Related to Oxidative Stress and Antibacterial Capacity?

**DOI:** 10.1021/acsomega.3c07702

**Published:** 2024-01-24

**Authors:** Ana Martínez, Isabel López-Rull

**Affiliations:** † Departamento de Materiales de Baja Dimensionalidad, Instituto de Investigaciones en Materiales, Universidad Nacional Autónoma de México, Circuito Exterior S. N. Ciudad Universitaria, CP 04510 Ciudad de México, México; ‡ Departamento Biología y Geología, Física y Química Inorgánica, Área de Biodiversidad y Conservación, Universidad Rey Juan Carlos, C/Tulipán s/n., Móstoles, Madrid 28933, Spain; § Instituto de Investigación en Cambio Global (IICG-URJC), C/Tulipán s/n., Móstoles, Madrid 28933, Spain

## Abstract

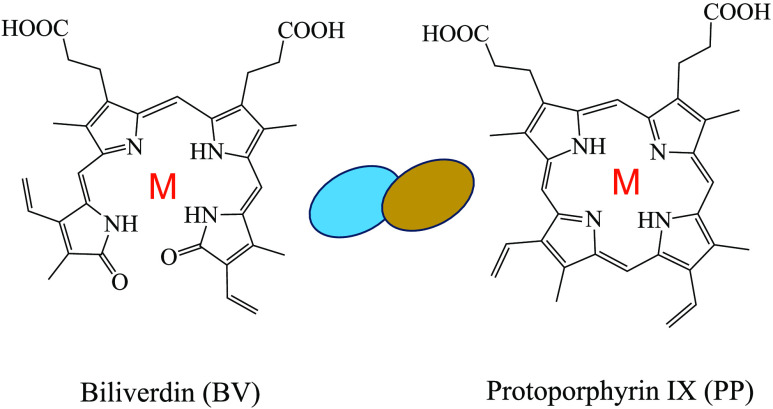

Two main substances are responsible for the pigmentation of the eggshells of birds and reptiles: the bluish-green tone comes from biliverdin (BV), and protoporphyrin IX (PP) gives brown hues. BV and PP can form complexes with metal cations. The main objective of this investigation is to carry out a theoretical study that analyzes the interaction of metal cations (Cu^2+^, Ag^2+^, Au^2+^, Cd^2+^, Zn^2+^, and Hg^2+^) with BV and PP. The divalent metal ions of Cu and Ag are selected to have compounds with the same charge. Density functional theory (DFT) calculations were used to investigate the antiradical capacity of these systems and to obtain ultraviolet–visible (UV–vis) spectra to analyze color modifications. Antiradical capacity is one of the mechanisms that prevents oxidative stress. The antibacterial capacity was investigated through the formation of triplet states. From our results, we can conclude that metal cations interacting with BV and PP affect the electron donor–acceptor properties of the systems, modify coloration of the eggshell, and increase the photoactivating capacity of pigments, which is related to their antimicrobial action. Electron transfer is an important mechanism of antioxidant defense. These results provide useful information on both the influence of contaminants such as heavy metals on the antimicrobial capacity of natural pigments and the signaling value of eggshell coloration.

## Introduction

Nature is colorful. Coloration occurs either through the scattering of light by tissues arranged on a nanometer scale or by pigments that absorb certain wavelengths of light.^1,2^ Pigmentation has many functions, from cryptic camouflage to aposematism and social signaling, thermoregulation, or parasite resistance.^3^ Hence, given that coloration is often associated with several fitness-related traits, it is a key aspect to understand animal evolution. There are many substances that produce a great variation of colors in animals and plants. Some pigments are related to sexual selection and survival due to their antioxidant or antibacterial properties. As an example, yellow, orange, and red pigmentation of animals, plants, and fruits are mostly produced by carotenoids.^1−5^ These substances are considered antioxidants or antiradicals that prevent oxidative stress.^6−13^ Animals do not produce carotenoids, they get them from food.^14,15^ Carotenoid-dependent traits are used as individual indicators of quality reflecting foraging ability, nutritional conditions, and antioxidant and immunological states in both vertebrates (mainly studied in birds and reptiles) and invertebrates (e.g., insects and crustaceans).^1^ Among the latter, the intensity of the pink-red coloration of crustaceans is related to the concentration of astaxanthin,^14^ an oxocarotenoid recognized as one of the best antiradical substances. The signal behind it is that “the redder is the healthier”.

**Figure 1:**
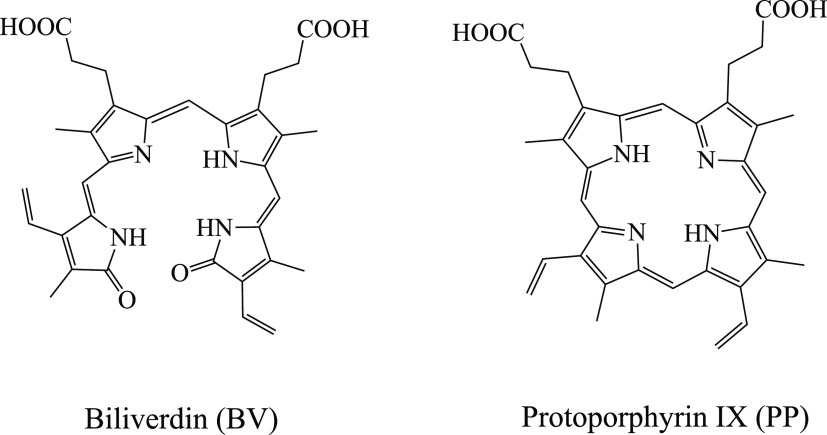
Schematic representation of the molecules under study.

Animal coloration can also be expressed beyond the immediate biological phenotype of individuals, as in the case of eggshells. Like carotenoid-colored body traits, eggshell coloration seems to fulfill multiple functions and has been related to the health conditions and genetic quality of females and also to egg internal quality in terms of yolk and albumen hormones and albumen antimicrobial proteins.^15−22^ Two main substances are responsible for the pigmentation of the eggshell of birds and reptiles: the bluish-green tone comes from biliverdin (BV), and protoporphyrin IX (PP) gives brown hues (see Figure 1 for molecular formulas). These substances are generated through the metabolism of the hemo group.^23,24^

BV and PP have been reported as being antiradical and antibacterial. As antiradical, one of the mechanisms that explain the ability to prevent oxidative stress is the electron transfer.^12,13^ Substances donate electrons to different free radicals such as OH^•^ or accept electrons from the superoxide radical anion (O_2_^•–^). As antimicrobial, the defense is through photoactivation, and the mechanism implies excited states.^26^ PP is photoactivated with sunlight and forms the triplet. The triplet excited state releases energy to the oxygen molecule, which then produces free radicals such as singlet oxygen. This free radical generates oxidative stress and can destroy some bacteria. Previous theoretical studies indicate that this mechanism might also be possible with BV.^20,24,25^

Environmental pollution with metals can significantly affect animal coloration as pigments may interact with metal cations.^27−31^ Density functional theory (DFT) studies concerning the formation of metal complexes with astaxanthin concluded that metal cations induce changes in the maximum absorption bands that are red-shifted.^27^ This theoretical study motivated experiments with shrimp (that have astaxanthin) exposed to copper, which turned redder in the presence of copper after being boiled.^31^ Such a relevant finding not only corroborates a prediction made from quantum chemistry calculations but also indicates that more red is not always healthier, possibly because the presence of metallic cations, which can be toxic, chelates carotenoids and produces redder colors. In this case, the red color is not a consequence of the amount of astaxanthin but the presence of heavy metals. A similar mechanism may occur in eggshell pigments. So far, the interaction of metal cations with eggshell pigments and also the consequences in the color. In such a situation, individuals displaying colorful eggshells could be misleadingly interpreted because such coloration would not necessarily signal individual quality. In fact, the signaling function of eggshell PP is ambiguous since it may signal both high antioxidative capacity of females when they are able to deposit these antioxidants into the eggshell during egg formation^21^ and also increased physiological stress and poor female condition due to the stress inductor properties of PP^32,33^ and the observation that it seems to elevate plasma heat shock proteins,^34^ the indicators of increased oxidative stress.^35^

In the case of shrimps, the redder individuals are more expensive in the market as people select them over less colorful individuals.^36−39^ The idea behind this is that the red color is related to the concentration of carotenoids that helps to reduce oxidative stress. The red color in the prawns tell us that it is food in good condition. However, this is not always the case since the presence of metal cations, which can be toxic, chelates carotenoids, and gives redder substances. In this case, the red color is not a consequence of the amount of astaxanthin but of the presence of heavy metals.

Previous results indicate that metal cations can react with BV.^28^ This research was related to the fluorescence of chelated compounds, seeking applications as materials for the diagnosis and treatment of diseases. There are also previous investigations of PP interacting with gold nanoparticles to generate useful materials for photodynamic therapy.^29^ Despite these results, color changes of BV and PP when interacting with metal cations remain uninvestigated: neither the influence of metals in the antioxidants nor antibacterial properties of pigments. Therefore, the main goal of this investigation is to carry out a theoretical study that analyzes the interaction between metal cations such as Cu^2+^, Ag^2+^, Au^2+^, Cd^2+^, Zn^2+^, and Hg^2+^ with BV and PP. The divalent metal ions of Cu and Ag are selected to have compounds with the same charge. In this research, the antiradical capacity of BV and PP interacting with metal cations (BV-M and PP-M) is analyzed. Ultraviolet–visible (UV–vis) spectra are obtained, and the antibacterial capacity is investigated through the formation of triplet states. These results provide useful information about the possible influence of contaminants (such as heavy metals) on animal signals that is relevant to the conservation of the species.

## Computational Details

**Figure 2:**
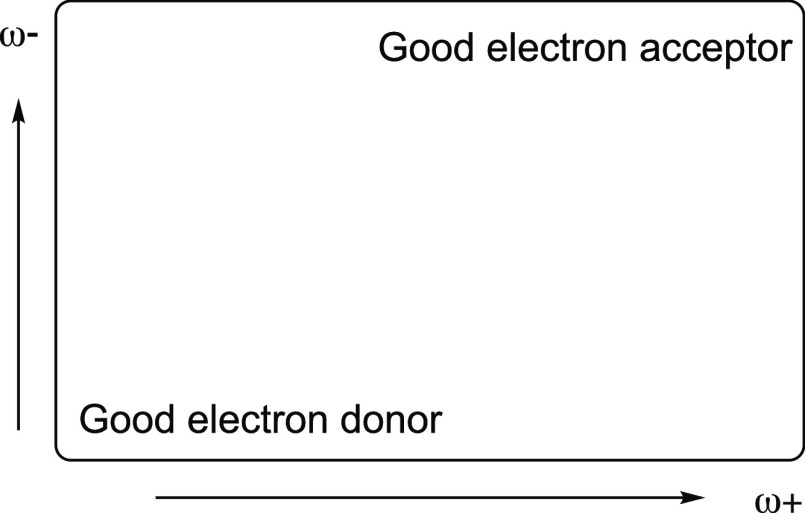
Electron Donor–Acceptor Map (DAM).

Gaussian09 was used for all electronic calculations.^40^ Geometry optimizations of initial geometries were obtained at M062x/LANL2DZ level of theory without symmetry constraints.^41−43^ LANL2DZ is a pseudopotential available for a variety of elements. These potentials have not been defined for the elements H–Ne. For these elements, all-electron double-ζ basis sets developed by Dunning (D95 V) are used. Harmonic analyses verified the local minima. The absorption spectra have been computed with time-dependent density functional theory (TDDFT) using single-point calculations of the optimized geometries at the same level of theory in water.^44−46^ For copper and zinc chelates, single-point calculations with the optimized geometries at the M062x/LANL2DZ level were calculated at the M062*x*/6-311+g(2d,p) level of theory. To obtain the excitation energies, single-point calculations of singlets and triplets were obtained at the same level of theory and with M062*x*/6-311+g(2d,p) for copper and zinc compounds. For the other metal cations, this base does not exist, so the calculations were made with LANL2DZ. Conceptual density functional theory is a chemical reactivity theory developed on density functional theory based concepts.^47−50^ Within this theory, there are response functions such as the electron-donating (ω^–^) and electron-accepting (ω^+^) powers previously reported by Gázquez et al.^48,49^ The capacity to donate electrons (ω^–^) and the propensity to accept electrons (ω^+^) are defined as follows
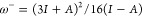
1
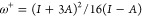
2*I* and *A* are vertical ionization energy and vertical electron affinity, respectively. Low values of ω^–^ indicate good electron donor molecules. High values of ω^+^ are good for electron acceptor molecules. These two quantities refer to charge transfers and not necessarily one electron. These chemical descriptors have been used successfully in different chemical systems.^51−54^ With these parameters, it is possible to determine the Electron Donor–Acceptor Map (DAM, see Figure 2).^12^ Systems located down to the left are considered good electron donors while those situated up to the right are good electron acceptors. *I* and *A* were obtained as follows
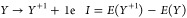
3
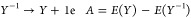
4

These values were calculated with single-point calculations of the optimized geometries at the M062x/LANL2DZ level of theory. For copper and zinc systems, we used 6-311+g(2d,p).

## Results and Discussion

**Figure 3:**
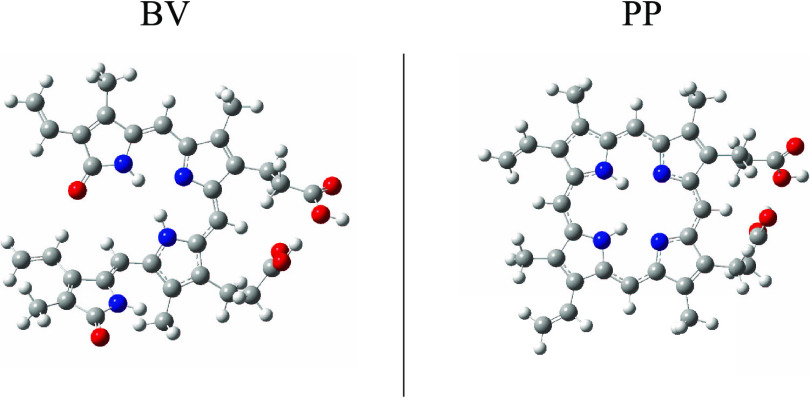
Optimized structures of biliverdin (BV) and protoporphyrin IX (PP). Red spheres represent oxygen atoms, blue spheres are nitrogen atoms, and gray spheres are carbon atoms.

BV and PP present structures with some similarities and important differences. Both are tetrapyrroles, but PP is a macrocycle. The only difference in their molecular formulas is that BV (C_33_H_34_N_4_O_6_) has two more oxygen atoms than PP (C_33_H_34_N_4_O_4_). The optimized geometries of these two molecules are shown in Figure 3. PP is more planar than BV and has four pyrrole groups that form a macrocycle. The presence of the two extra oxygen atoms causes one of the pyrrole groups of BV to be out of plane. The other three are located forming a nonflat quasi-cycle. This difference is important for the interaction with metals.

**Figure 4:**
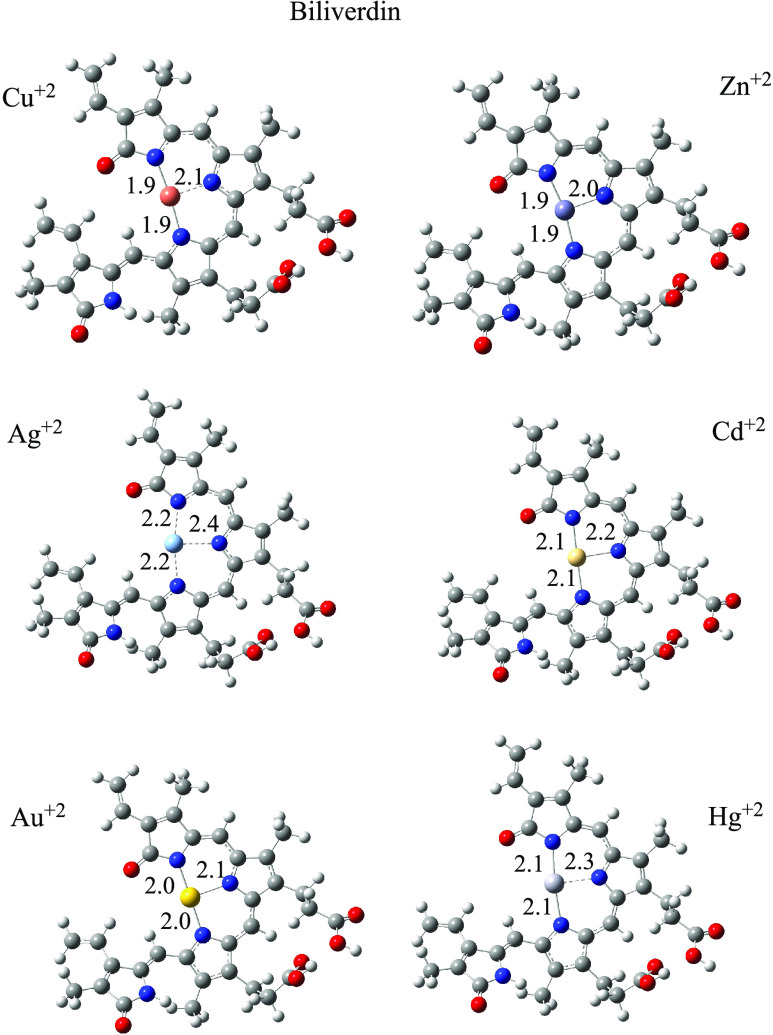
Optimized structures of BV-M. Bond distances are in Å. Red spheres represent oxygen atoms, blue spheres are nitrogen atoms, and gray spheres are carbon atoms.

**Figure 5:**
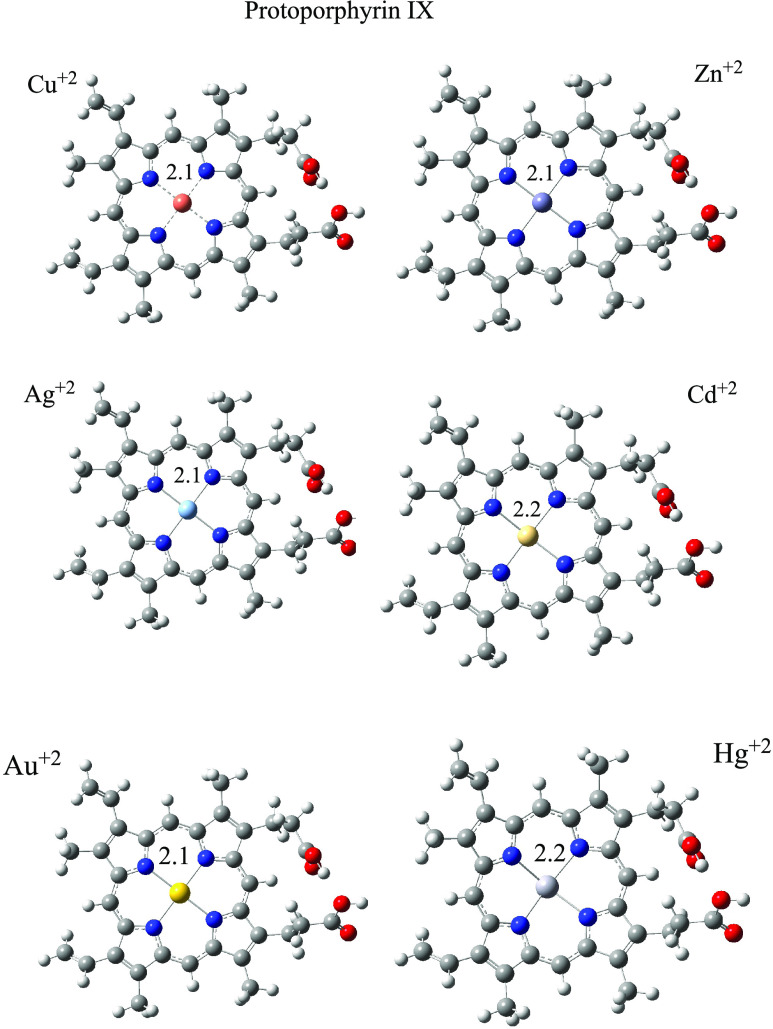
Optimized structures of PP-M. Bond distances are in Å. Red spheres represent oxygen atoms, blue spheres are nitrogen atoms, and gray spheres are carbon atoms.

To analyze the interaction between BV and PP with metal cations, we remove two protons from two pyrrole groups in both cases, and we obtain neutral systems. Optimized structures of compounds with metals are shown in Figures 4 and 5. BV forms three N–M bonds, and PP forms four N–M bonds. Around metal cations, the structures are planar and bond distances are similar. For BV, there are two N–M bond distances that are equal and there is one that is greater in all structures. The four M–N bond lengths of the PP systems are all the same. Cd^2+^ and Hg^2+^ with BV and PP have longer M–N bond distances than with the other metals. The exception is BV–Ag that presents the longest M–N bond length.

**Figure 6:**
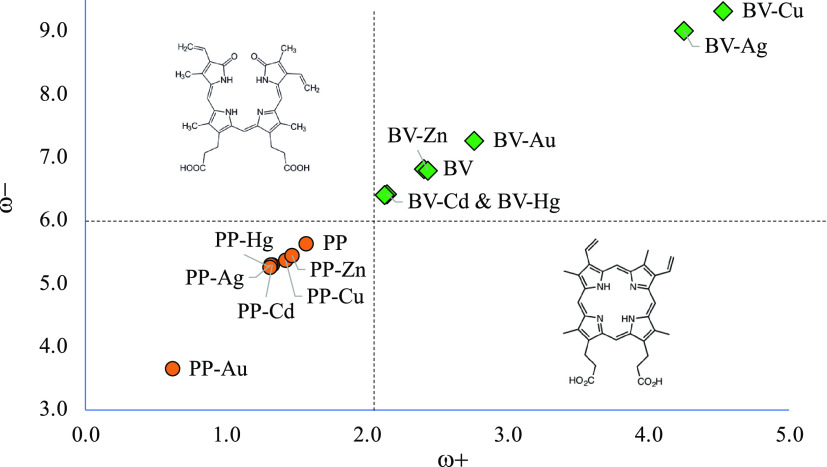
Electron Donor–Acceptor Map (DAM) of the compounds under study. Values in eV.

The free-radical scavenger capacity of BV and PP was previously reported^21^ according to the electron transfer capacity. It was concluded that BV is a good antiradical since it is a good electron acceptor that is able to inactivate the superoxide anion (O_2_^•–^), and PP is as good electron donor as some yellow carotenoids that are considered good antioxidants. To analyze the effect of the metals in the electron transfer capacity, in Figure 6, we report the DAM of the studied compounds.

The first thing to notice is that there are two sections: one for PP systems and the other for BV compounds. It can be considered that PP systems are good electron donors (antioxidants), and BV systems are good electron acceptors (antireductants). With BV, compounds with Cu^2+^ and Ag^2+^ are the best electron acceptors. They are better than BV, and BV–Au is also a better electron acceptor than BV. With Cd^2+^ and Hg^2+^, systems are worse electron acceptors and better electron donors than BV. The electron transfer capacity of BV–Zn is equal to the capacity of BV.

Concerning PP systems, they have a similar electron donor–acceptor capacity, PP being the worse and PP-Au being the best electron donor. PP is the best electron acceptor among the compounds with PP. Molecules with PP are better electron donors than BV systems, but the differences in these systems are smaller than in the case with BV.

With these results, it can be said that the electron donor–acceptor properties are modified in the presence of metal cations, in addition that Cu^2+^ and Ag^2+^ have a greater influence on the properties of BV, and Au^2+^ modifies the ability to donate electrons of PP. It remains to be established whether this heavy metal-mediated change in the antiradical properties of eggshell pigments may affect embryo development and survival. It has been recently proposed that avian embryo may gradually absorb pigment traces from the eggshell, as it does with calcium, and that pigments may have the potential to directly promote embryo survival due to antioxidant and antiradical capacities.^24^ Future empirical research on the study of the presence of BV and PP traces in the inner eggshell layers and the link of these traces with embryonic exposure to oxidative damage mediated by pollution with heavy metals would be necessary to confirm our observed heavy-metal-mediated change in the antiradical properties of eggshell pigments.

**Figure 7:**
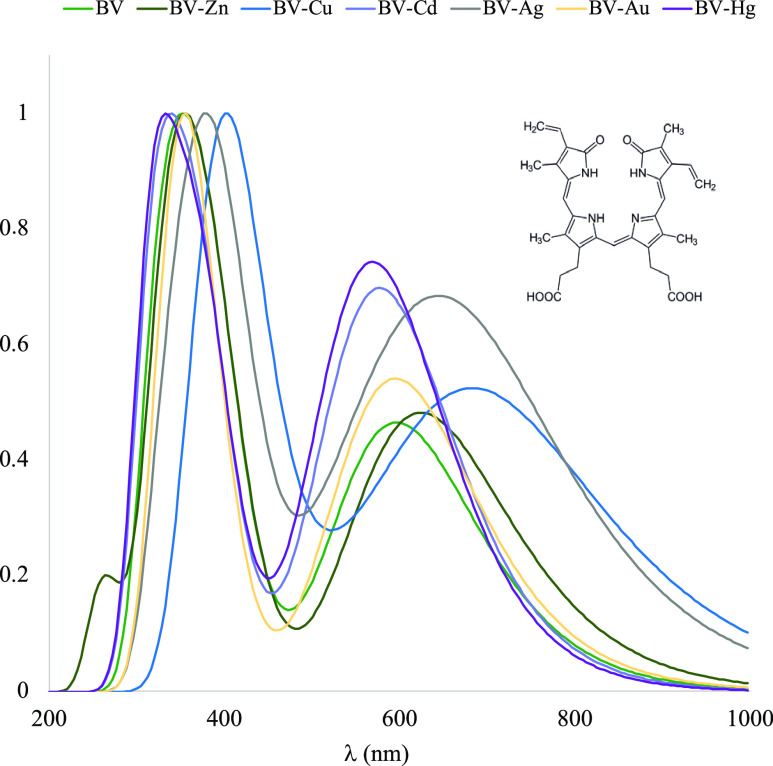
UV–visible spectra calculated in water of the compounds with BV.

**Figure 8:**
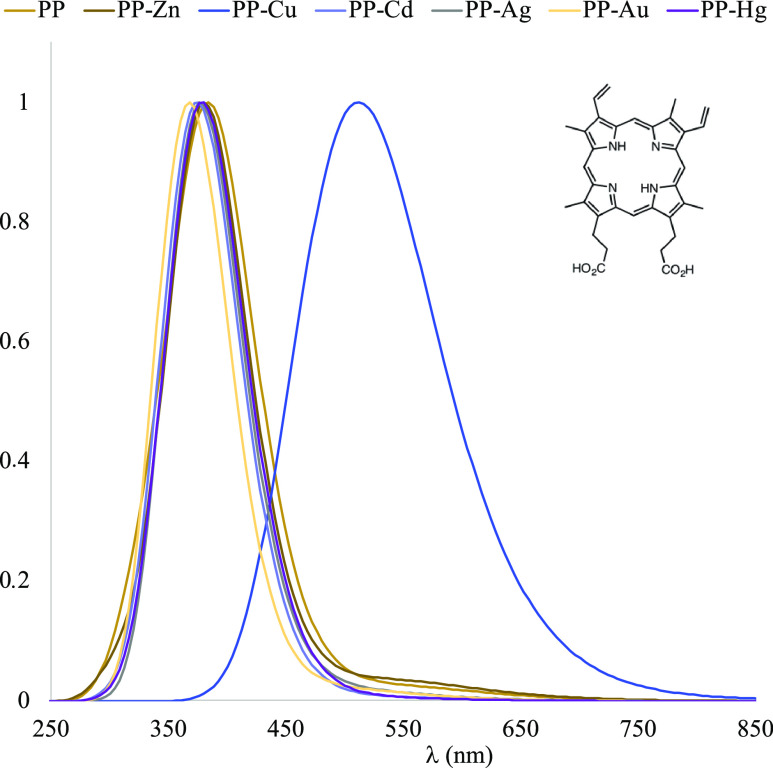
UV–visible spectra calculated in water of the compounds with PP.

To analyze the changes in color due to the presence of metal cations, UV–visible spectra were obtained. The results are reported in Figures 7 and 8. The values of λ_max_ are summarized in Table 1 for immediate reference.

**Table 1:** Values of λ _max_ Calculated in Water^a^

compound	λ_max_ (nm)		compound	λ_max_ (nm)
BV	352	596	PP	384
BV–Cu	404	684	PP-Cu	512
BV–Ag	380	644	PP-Ag	376
BV–Au	356	596	PP-Au	368
BV–Zn	356	624	PP-Zn	380
BV–Cd	340	576	PP-Cd	376
BV–Hg	332	568	PP-Hg	380

a The systems with BV present two maxima.

For BV with metal cations, spectra and λ_max_ values are similar but the presence of M^+2^ produces signals at higher wavelengths. The exception is with Au^2+^ since BV and BV–Au present the same values. BV signals are more sensitive to the presence of metal cations such as Cu^2+^, Ag^2+^, and Zn^2+^, with BV–Cu being the system that presents the greatest changes in the UV–visible spectrum. For PP, the spectra and λ_max_ values are similar but the presence of M^+2^ also produces higher wavelengths than for PP. The presence of Cu^2+^ generates the biggest modifications, as PP-Cu is the system with higher wavelength in the UV–visible spectra. Similar results were found with carotenoids in a theoretical study^23^ and also in an experiment with shrimps.^27^ However, there are no experimental studies or observations showing that the interaction of metal cations with eggshell pigments can produce more intense colors. Our theoretical results in the field remain to be confirmed. Changes in eggshell color due to the interaction of metal cations with pigments would be interesting in relation to social signaling, since both BV and PP have been proposed to function as a postmating sexually selected signal of female quality, either good quality in the case of BV or good/bad quality, as we mentioned above for PP. Regardless of the direction of the signal, our finding may imply that individuals displaying colorful eggshells could be misleadingly interpreted because such coloration may be due to contamination with metals. With the results reported here, it is possible to say that changes in the color of the eggshell indicate the presence of metal cations. Although more observations are needed to experimentally corroborate these findings and their consequences, these results are important and may be helpful in the design of new experiments.

**Figure 9:**
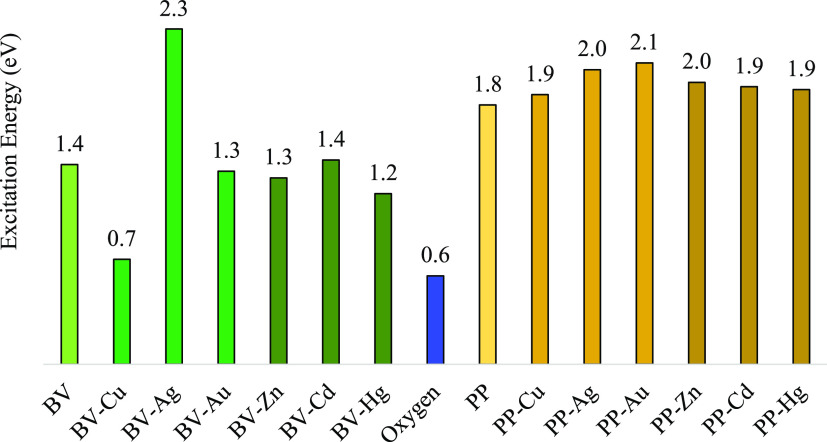
Excitation energies of the systems under study.

The last question that we would like to answer is related to the antibacterial defense through photoactivation, which implies excited states. We calculated the excited states of all compounds (triplets for BV, PP and the systems with Zn^2+^, Cd^2+^, and Hg^2+^; and quadruplets for systems with Cu^2+^, Ag^2+^, and Au^2+^). The results are reported in Figure 9. The photoactivation mechanism suggested is as follows: BV or PP is excited by sunlight; these excited states release excess energy when they return to ground states; this released energy produces singlet oxygen. Singlet oxygen excited state is highly reactive and contributes to the production of free radicals. Free radicals produce oxidative stress in bacteria and pathogens until they die. It is important to note that BV and PP are found on the surface of the eggshell. Photoactivation and production of singlet oxygen occur outside the egg, and this explains why singlet oxygen does not affect the proteins of the embryo and does not alter its development.

According to this mechanism, the excitation energy that is released must be enough to produce the excitation of molecular oxygen to produce the singlet oxygen. Figure 9 indicates that the excitation energy of molecular oxygen is 0.6 eV. All values for different systems with BV and PP have higher excitation energies and therefore are able to produce singlet oxygen. However, for BV–Cu, the excitation energy is 0.7 eV, which is close to the value of molecular oxygen and can be considered within the limits of the calculation. Therefore, it can be expected that BV–Cu does not show photoactivated antimicrobial action like the other compounds. Comparing the values of Figure 9, it is possible to observe that the excitation energies of the systems with PP are higher than the excitation energies of the BV systems, with the exception of BV–Ag, which presents the highest value. This means that PP-M and PP are better antimicrobial than BV-M and BV. These results are in agreement with previous results for gold in which PP-Au nanoparticles were reported^25^ to be efficient photosensitizers in cervical cancer therapy, better than PP. The authors reported that one possible explanation is that PP-Au has higher excitation energy than PP. The PP-M potentiation of the photoactivating capacity that we found suggests an increase in the photoactivating capacity of PP, which is related to the antimicrobial action. These results must be tested in the field, but they indicate that the presence of metal cations could promote embryo survival in birds and reptiles due to the inactivation of pathogens since a main source of mortality for early life stages of oviparous vertebrates is microbial infection of eggs.

Summarizing, our results indicate that the presence of metal cations interacting with BV and PP affects the electron donor–acceptor properties of the systems, modifies eggshell coloration, and increases the photoactivating capacity of pigments, which is related to their antimicrobial action. The consequences of this on the development and survival of embryos have not yet established. More observations are needed to experimentally corroborate these results and their consequences for embryonic survival and avian egg coloration in a signaling context.

## Conclusions

Metal cations may interact with BV and PP to form chelated compounds. The electron transfer properties are modified with the presence of metallic cations, and Cu^2+^ and Ag^2+^ have a greater influence on the properties of BV. The systems with BV are antireductants (electron acceptors) and PP systems are antioxidants (electron donors). BV–Cu and BV–Ag are the best electron acceptors, and PP-Au is the best electron donor. These electron transfer properties prevent the oxidative stress and are an indication of the possible effects that heavy metals may produce.

For PP, the spectra and λ_max_ values are similar but the presence of M^+2^ also produces higher wavelengths than for PP. The presence of Cu^2+^ generates the greatest modifications, with BV–Cu being the system with the longest wavelength in the UV–visible spectrum. Similar results were found with carotenoids in a theoretical study and also in the experiment with shrimps.

Excitation energies of systems with PP are higher than those of BV systems, with the exception of BV–Ag, which presents the highest value. This means that PP and PP-M may be better antimicrobials than BV and BV-M.
